# Keeping up with the nicotinamides: NADP(H), the forgotten circadian cofactor that keeps metabolic time

**DOI:** 10.1093/lifemeta/loaf034

**Published:** 2026-04-20

**Authors:** Lauren Palluth, Joseph S Takahashi, Carla B Green

**Affiliations:** Department of Neuroscience, Peter O’Donnell Brain Institute, University of Texas Southwestern Medical Center, Dallas, TX 75390, the United States; Department of Neuroscience, Peter O’Donnell Brain Institute, University of Texas Southwestern Medical Center, Dallas, TX 75390, the United States; Department of Neuroscience, Peter O’Donnell Brain Institute, University of Texas Southwestern Medical Center, Dallas, TX 75390, the United States

**Keywords:** NADP(H), circadian, metabolism, nocturnin, NAD kinase, NAMPT

## Abstract

The hierarchical relationship between the core circadian clock of the suprachiasmatic nucleus and peripheral clocks throughout the body is tightly regulated. Nicotinamide adenine dinucleotide phosphate (NADP(H)) is a rhythmic cofactor used in hundreds of metabolic reactions. The cellular NADP(H) pool is not only regulated by several clock-controlled enzymes, but also responsive to peripheral “zeitgebers” such as food intake and oxidative stress. This positions NADP(H) as a potential harbinger between core and peripheral metabolic rhythms. While discussion in recent years has focused on its unphosphorylated counterpart, NAD(H), this review aims to highlight the roles of NADP(H) in circadian metabolism. This review discusses the multilayered regulation of cellular NADP(H), how the total pool size, redox ratio, and rhythmicity of NADP(H) impact core and peripheral rhythms, and how disruption of its rhythmic regulation can lead to metabolic disease.

## Introduction

**Figure 1:**
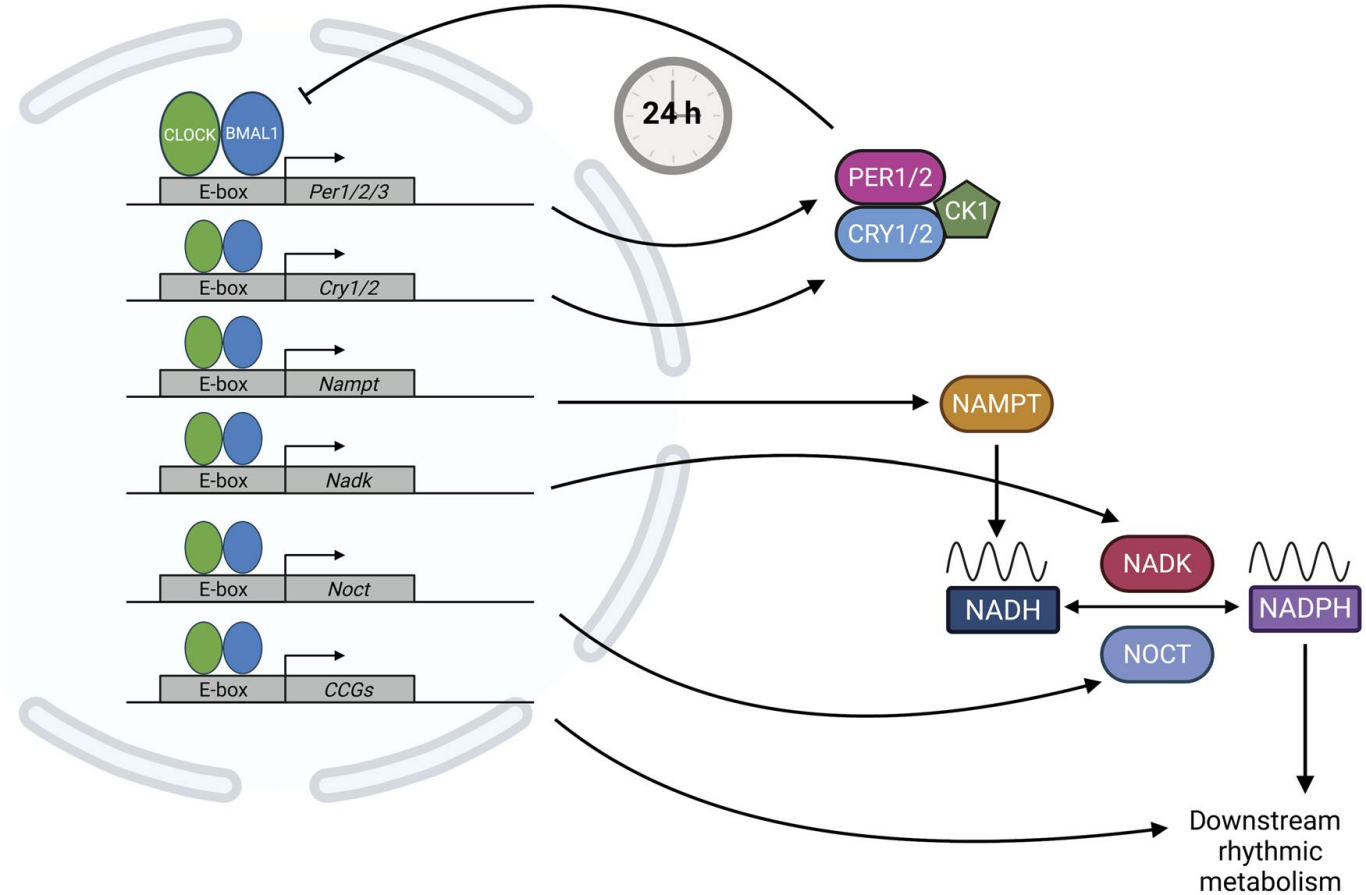
Core clock TTFL and its regulation of NADP(H). The core TTFL is driven by the transcription factors CLOCK and BMAL1 that heterodimerize and bind to the E-box regions of DNA to promote the transcription of *Per1/2* and *Cry1/2*. PER1/2 and CRY1/2 proteins accumulate in the cytoplasm and form a complex with casein kinase 1 alpha and epsilon (CK1δ/ε). This complex then translocates to the nucleus and represses the activity of the CLOCK/BMAL1 heterodimer. The full activation and repression phases take approximately 24 h to complete. CLOCK and BMAL1 bind to other E-box regions of the DNA, promoting the transcription of other genes, including *Nampt*, *Nadk*, and *Noct*, which all regulate the cellular NADP(H) pool [12].

The temporal regulation of metabolism is an often-overlooked feature of the field. However, biological rhythms have been observed for centuries [1]. Organisms have evolved 24-h behavioral, physiological, and biochemical rhythms, termed circadian rhythms, that align with the Earth’s rotation. Over the last few decades, the molecular players driving circadian rhythms have been identified through a series of seminal mutagenesis screens [2–10]. It is now understood that these players make up a core transcriptional-translational feedback loop (TTFL) and activate the transcription of period (*Per*) and cryptochrome (*Cry*) genes, whose products, in turn, form a complex that inhibits the activity of the circadian locomotor output cycles kaput (CLOCK) and the brain and muscle ARNT-like 1 (BMAL1) [11, 12] (Fig. 1). Elegantly, this transcriptional activation and subsequent repression take roughly 24 h to complete. Notably, CLOCK and BMAL1 not only regulate the transcription of their own repressors, but also control the expression of thousands of other genes, generating robust metabolic rhythms [13].

An interesting feature of the circadian system is its hierarchical organization. It was discovered that the suprachiasmatic nucleus (SCN) contains the central pacemaker that entrains peripheral clocks [14, 15]. However, work has also shown that peripheral rhythms are responsive to other stimuli, including hormonal signaling and food intake [16–19]. Interestingly, before the core TTFL was discovered, many believed that energy metabolism was the sole driving force of all biological rhythms [20, 21]. In fact, some proposed that the pyridine dinucleotide cofactors, NAD^+^ and NADPH, alongside NAD^+^ kinase and NADP^+^ phosphatase, were key “gears” of the core clock, as they were some of the first metabolites found to rhythmically oscillate in free-running cultures of *Euglena* [22]. While it is now known that NADP(H) is not part of the core clock, it is still a key metabolite connecting central and peripheral oscillators. This review aims to highlight the role of NADP(H) in circadian metabolism and how NADP(H) serves as a mediator between central and peripheral rhythms.

## Introduction to NAD(H) and NADP(H)

Nicotinamide adenine dinucleotide (NAD(H)) and its phosphorylated counterpart (NADP(H)) are major regulators of metabolism. While they are mostly known as electron carriers that participate in redox biology, they are, in fact, some of the most ubiquitous metabolites in biology, with only water and protons exceeding their usage [23]. NADPH is known both for generating reactive oxygen species (ROS) through the NADPH oxidase (NOX) family of enzymes and for protecting the cell from ROS via the regeneration of reduced antioxidants [24]. NADPH also provides reducing power for a number of biosynthetic reactions, including but not limited to the synthesis of purines and fatty acids [25]. While NADPH is mainly involved in anabolism, NAD^+^ primarily works on the opposing catabolic arm of metabolism as the major electron carrier in glycolysis and the tricarboxylic acid (TCA) cycle. However, NAD^+^ is also involved in the deacetylation and ADP-ribosylation of proteins and nucleic acids via the NAD^+^-dependent sirtuin (SIRT) and poly (ADP-ribose) polymerase-1 (PARP-1) enzymes, respectively [26–28]. Additionally, NAD^+^ and NADP^+^ can serve as substrates for the synthesis of signaling molecules. Notably, NAD^+^ can be converted into cyclic ADP-ribose (cADPR), and NADP^+^ can be converted to nicotinic acid adenine dinucleotide phosphate (NAADP^+^), both of which participate in intracellular calcium signaling [29–32].

Given their widespread roles in metabolism, it is not surprising that the total levels, redox ratios, and subcellular localization of the pyridine dinucleotides are tightly regulated. In fact, the levels of both NAD(H) and NADP(H) robustly oscillate over the course of the day. This rhythmicity is primarily driven by the core circadian clock yet is also impacted by secondary “zeitgebers”, such as the nutrient and redox status of the cell [22, 33–36]. This, in effect, allows NADP(H) and NAD(H) to serve as major regulators of peripheral rhythms that likely temporally control hundreds of reactions that utilize them [33, 37, 38]. In fact, a quarter of the NAD(P)(H)-dependent enzymes in the liver exhibit circadian activity [23]. Therefore, NAD(H) and NADP(H) are particularly important metabolites controlling the interplay between circadian rhythms and metabolism.

While the pool sizes and redox ratios of NAD(H) and NADP(H) have yet to be strictly defined across different tissues, it is generally believed that cellular NAD(H) concentrations exceed that of NADP(H) [23]. This, coupled with the increasing fascination of NAD(H)’s role in fasting and longevity, has led recent research in both circadian and metabolic fields to focus on NAD(H) and focus less attention on the phosphorylated cofactor NADP(H). Therefore, the information synthesized in this review intends to highlight NADP(H)—from its roles in metabolism to redox balance to circadian rhythms. This review aims to not only underscore the importance of NADP(H) but also to encourage future research on NADP(H) in the realm of circadian metabolism.

### Synthesis and regulation of NADP(H) pool

NADP(H) can only be generated from NAD(H); therefore, the cellular NADP(H) pool is dependent on the synthesis of NAD(H). NAD^+^ can be synthesized from dietary tryptophan via the kynurenine pathway or dietary nicotinic acid via the Priess-Handler pathway. Most commonly, however, NAD^+^ is made through the salvage pathway, which recycles NAD^+^ precursors generated from NAD^+^-consuming reactions [39, 40]. Notably, nicotinamide phosphoribosyltransferase (NAMPT), the rate-limiting enzyme in the salvage pathway, displays robust circadian rhythmicity at both the RNA and protein levels in mouse liver. With this, it has been shown that CLOCK and BMAL1 directly bind to the *Nampt* promoter and regulate its expression, and NAMPT rhythmicity is completely abolished in clock mutant models [41–43] (Fig. 1). In fact, data suggest that modulation of different core clock components has differing downstream effects on NAMPT levels. Specifically, one study found that *Bmal1*^*−/−*^ and *Clock*^*Δ19*^ mice have decreased *Nampt* expression, and *Cry1/Cry2^*−/−*^* mice display increased *Nampt* expression in liver tissue [41]. This suggests that loss of the positive arm of the clock downregulates *Nampt* while loss of the negative arm upregulates its expression. However, another study found that loss of both reverse strand of ErbA alpha (*Rev-erbα*) and reverse ErbA beta (*Rev-erbβ*), other components of the negative arm of the clock, decreases *Nampt* expression and NAD^+^ levels in cardiomyocytes [44]. Interestingly, this study found that loss of *Rev-erb* derepresses the transcriptional repressor E4 promoter-binding protein 4 (E4BP4), which also regulates *Nampt* expression. Overall, this suggests that regulation of *Nampt*, and downstream NAD(H) and NADP(H) levels, by the core clock is multilayered. Reciprocally, however, NAMPT does not have any direct impact on the core clock. Muscle- and adipose-specific *Nampt*^*−/−*^ mice showed little to no disruption in circadian gene expression in the respective tissues [45]. This suggests that while NAMPT is a rhythmic output of the core clock, it does not invoke any significant feedback mechanism on such. NAMPT, however, is the primary driver of cellular NAD^+^ rhythms, for loss of *Nampt* damps NAD^+^ oscillations [41, 43, 45]. While NAMPT is circadian under constant conditions, NAD^+^ displays a bimodal ultradian oscillation in murine liver tissue, with peaks in the middle of the active and rest phases [41]. A similar bimodal pattern of NAD(H) levels was observed in both the cytosolic and mitochondrial fractions of rat liver tissue. However, the cytosolic and mitochondrial NAD(H) rhythms display different phases, suggesting that the temporal control of NAD(H) may differ across cellular compartments [34]. NADP(H) also rhythmically oscillates, which has been observed from algae to mammalian models, yet reports have found that NADP(H) rhythms have a circadian profile with only one peak during a day as opposed to two peaks of NAD(H) [22, 46, 47]. While it has been shown that NADP^+^ levels are lowered by inhibition of NAMPT, no study to date has examined how the rhythmicity of NADP(H) is affected by loss of NAMPT [48].

Similar to NAMPT, disruption of circadian gene expression impacts cellular NAD(H) levels. Lower NAD(H) concentrations were found in *Bmal1*^−/−^ and cardiomyocyte-specific *Rev-erb*-DKO mice, and higher NAD^+^ levels were found in *Cry1/Cry2*^−/−^ mice [33, 41, 44]. While one could hypothesize that NADP(H) would be affected by clock disruption in a similar manner, the impact on NADP(H) has not been studied. In fact, it is important to note that these studies only looked at NAD(H) levels at two time points, and no study to date has directly analyzed the impact of core clock disruption on NAD(H) nor NADP(H) rhythms across a 24-h period. Given that core clock mutants display an array of metabolic phenotypes and notable defects in NADP(H)-consuming pathways, such as lipid and sterol metabolism, more studies examining the interplay between NADP(H) and the core clock are needed [49–51].

### Regulation of NADP(H) through NADK

The rhythmicity of NADP(H) is not only indirectly impacted by the rhythmic generation of NAD^+^ via the NAMPT salvage pathway but also by the rhythmic activity of NAD kinase (NADK) [23] (Fig. 1). The activity of NADK was first observed in yeast homogenates in 1937 [52]. Subsequent studies have thoroughly characterized NADK in yeast and plants, both of which have three isoforms that differ in their subcellular localization [53]. The first human NADK was not cloned until decades later [54]. While the activity of human NADK was believed to be largely specific to the cytosol, there was speculation that another mammalian isoform existed, given the multiple isoforms found in lower species [55, 56]. In time, the protein C5orf33 was identified as a mitochondrial NADK, encoded by a separate gene than that of the cytosolic NADK, and was later renamed as NADK2 [57]. Notably, while NADK is rhythmically expressed, NADK2 does not appear to be under circadian control [13, 23]. The rhythmicity of different isoforms in plants and yeast has not been analyzed, but further studies are needed to understand the relationship between NADK’s rhythmic activity and subcellular localization.

### Regulation of NADP(H) through nocturnin

The conversion of NADP(H) to NAD(H) is also temporally regulated by the circadian NADP(H) phosphatase nocturnin (NOCT) (Fig. 1). NOCT was first discovered using mRNA differential display to screen for rhythmic transcripts in *Xenopus laevis* retina [58]. As NOCT was identified in the same years that the core clock TTFL was being characterized, NOCT was first believed to be a candidate component of the TTFL or at least an effector of clock function. This was not necessarily an inane hypothesis, for NOCT is robustly rhythmic with a > 40-fold increase in transcription from its trough in the early day to its peak at the onset of night (ZT12) [59]. NOCT was found to be both present and rhythmic in nearly all murine tissues, and its robust oscillation persists in constant dark (DD) conditions [60]. While it was later found that NOCT is not in fact a key component in the core TTFL, its role as a downstream effector of peripheral rhythms has become increasingly apparent. Studies first examining the transcriptional control of *Xenopus Noct* found that *Noct* does not have a canonical E-box in its promoter region [61, 62]. Rather, it contains what was then coined as the nocturnin element (NE), which differs from canonical E-boxes by just one base pair [62]. This study found that while CLOCK fails to bind to the NE, cAMP response element-binding protein (CREB) could bind to the NE and promote the rhythmic transcription of *Noct* [62]. Other studies examining the transcriptional regulation of *Noct* in mammals found that the promoter region of the mammalian *Noct* gene differs significantly from that of *Xenopus* and does, in fact, contain two canonical E-box regions. It was then found that CLOCK and BMAL1 bind to both E-boxes, with a strong preference for the second E-box region, and, in turn, regulate *Noct* transcription [63]. While this study did not determine whether CREB also regulates mammalian *Noct* like that of *Xenopus*, it did highlight that mammalian *Noct* promoter contained eight CREs and five Reverbα-binding sites (ROREs), suggesting that NOCT may be temporally controlled by several mechanisms. This layered regulation may explain why *Noct* appeared to remain rhythmic, albeit slightly damped, in two different clock mutant systems [64, 65].

Given the sequence similarity of NOCT to the C-C motif chemokine receptor 4 (CCR4)-like family of proteins, it was first believed that NOCT functioned as a deadenylase [66]. While NOCT did show deadenylase activity at saturating concentrations *in vitro*, it was later proven that NOCT primarily acts as an NADP(H) phosphatase [67–69]. In characterizing the function of NOCT, one study found that the addition of purified human NOCT to either bovine liver or HEK293T cell lysates specifically depleted NADP^+^ and NADPH, and reciprocally increased NAD^+^ and NADH levels. This was further confirmed in A549 cells, where *Noct*-KO significantly increased the cellular NADP(H)/NAD(H) ratio [68]. Additionally, a separate study found that overexpression of *Noct* in HEK293 cells significantly decreased cellular NADP(H) levels [70]. The discovery that NOCT functions as a NADP(H) phosphatase is significant, for the only other known NADP(H) phosphatase is metazoan SpoT homolog-1 (MESH1), about which very little is known [71]. More studies are needed to both better characterize MESH1 and differentiate its role from NOCT.

NOCT has two isoforms that differ in their translational start sites. If translation initiates at the first methionine, a mitochondrial targeting sequence is translated, and NOCT localizes to the mitochondria [68, 70, 72, 73]. However, if translation initiates at the second methionine, a shorter isoform without a targeting sequence is translated, and NOCT resides in the cytoplasm and associates with the endoplasmic reticulum (ER) due to its N-terminal myristoylation [70]. Interestingly, the two isoforms exhibit differences in rhythmicity. While this is similar to the difference in rhythmicity seen between NADK and NADK2, the mitochondrial form of NOCT is highly rhythmic, whereas the cytosolic isoform is constitutively expressed. This high-amplitude diurnal variation of mitochondrial NOCT is especially interesting given that NOCT is the only known mitochondrial NADP(H) phosphatase. While MESH1 is the only other identified cytosolic NADP(H) phosphatase, its rhythmicity, or lack thereof, has yet to be investigated [71]. Further, while the impact of NOCT on total NADP(H) levels has been measured, its impact on NADP(H) rhythmicity has yet to be reported.

### Regulation of NADP^+^/NADPH redox ratio

**Figure 2:**
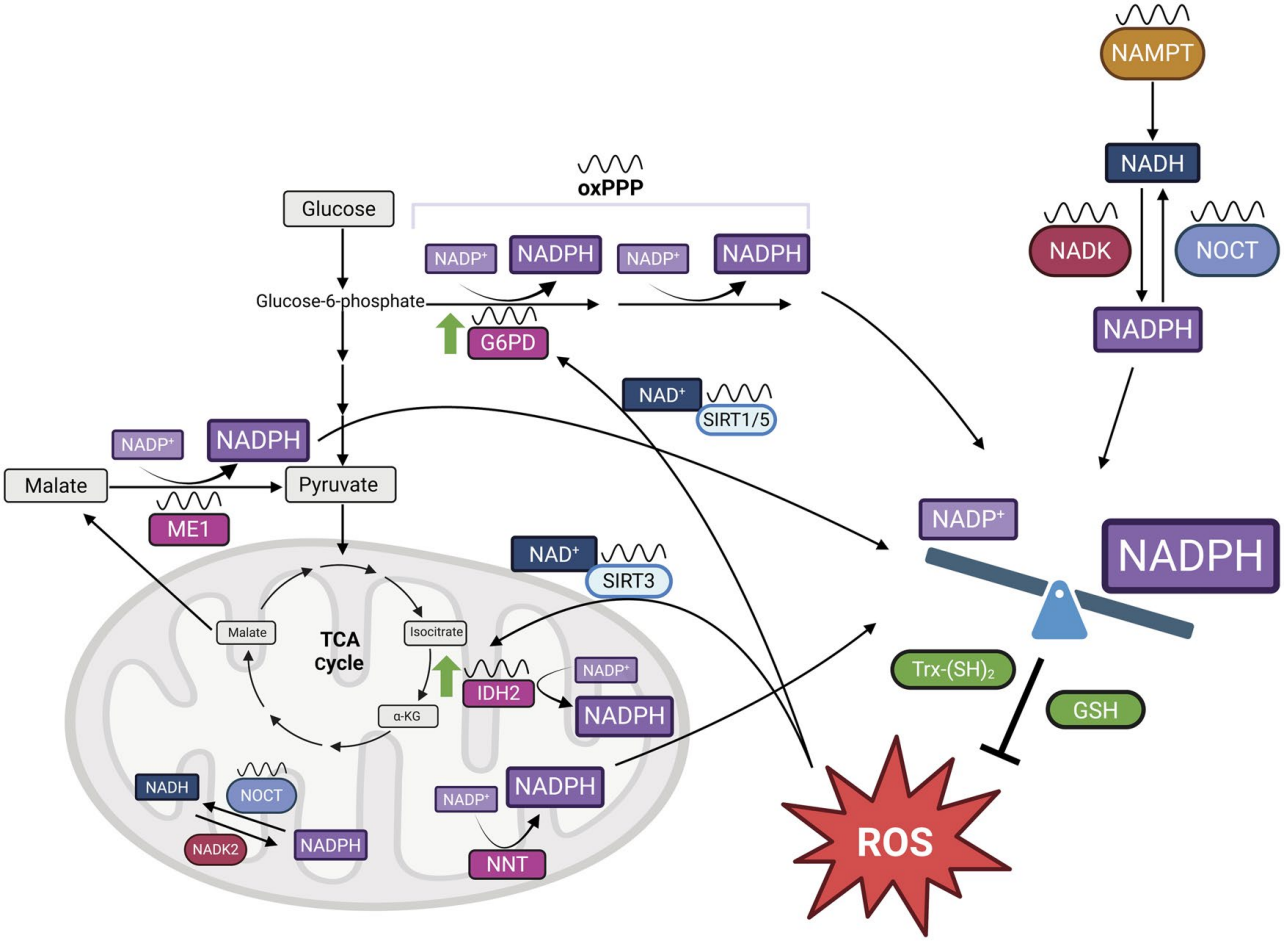
The rhythmic regulation of the NADP^+^/NADPH redox ratio in response to ROS. Rhythmic enzymes G6PD, ME1, and IDH1 can convert NADP^+^ to NADPH. In response to ROS, circadian sirtuin enzymes, specifically SIRT1/5 and SIRT3, can use NAD^+^ to deacetylate and activate G6PD and IDH2, respectively, to decrease the cellular NADP^+^/NADPH redox ratio. NAMPT, the rate-limiting enzyme in NAD(H) synthesis, and NADK and NOCT, which control the interconversion of NAD(H) and NADP(H), are circadian controlled. Within the mitochondria, NOCT also rhythmically regulates NADP(H) levels, and NNT can convert NADP^+^ to NADPH. Reduced NADPH can regenerate the reduced antioxidant enzymes, GSH and Trx-(SH)_2_, to help combat ROS [74].

In addition to the overall pool size, the redox state of NADP(H) is also tightly regulated by several metabolic pathways [74] (Fig. 2). The major sources of cytosolic NADPH are the oxidative pentose phosphate pathway (oxPPP), cytosolic isocitrate dehydrogenase (IDH1), and cytosolic malic enzyme (ME1) [75]. In most cell types, the oxPPP is the main contributor to cytosolic NADPH, as both glucose-6-phosphate dehydrogenase (G6PD) and 6-phosphogluconate dehydrogenase (6PGD) reduce NADP^+^ to NADPH [75]. There is increasing evidence, however, that the source of NADPH may vary across tissues as well as metabolic states. For instance, one study found that ME1 is the major source of NADPH in differentiating adipocytes under normoxic conditions. However, under hypoxic conditions, NADPH is primarily produced by the oxPPP [76]. It is also believed that NADPH produced by these different pathways has different fates. While reports suggest that the oxPPP activity directly parallels lipogenesis, others found that fatty acid synthesis is maintained in *G6PD*^*−/−*^ cell lines [75, 77]. Loss of G6PD, however, significantly impairs downstream folate metabolism [75]. This again may differ across tissues, for one study found that the oxPPP fuels lipogenesis in brown adipose tissue yet serine catabolism is the major driver of lipogenesis in liver tissue [78]. Interestingly, evidence suggests that the ­oxPPP and ME1 are circadian, making it likely that even the NADP^+^/NADPH redox ratio temporally controlled [37, 70, 79].

NADP^+^/NADPH regulation also differs by cellular compartment. Within the mitochondria, folate metabolism, mitochondrial isocitrate dehydrogenase (IDH2), mitochondrial malic enzyme (ME3), glutamate dehydrogenase, and nicotinamide nucleotide transhydrogenase (NNT) are main producers of NADPH [39, 80–84]. In *E. coli*, NNT produces almost half of cellular NADPH, leading to the belief that NNT is the major mitochondrial source of NADPH [85]. While this has not been directly measured in eukaryotes, it has been shown that *Nnt* mutation leads to a significant reduction in NADPH as well as an impaired oxidative stress response [84, 86]. This is especially notable given the widespread use of C57BL/6N and C57BL/6J mice, for one of the major differences between the two strains is a mutation in *Nnt* in the C57BL/6J strain but not the C57BL/6N strain [87]. Little is known about whether the activity or expression of NNT is rhythmically regulated. Given the use of both C57BL/6J and C57BL/6N mice in both metabolic and circadian research, studies examining the differences in NADP(H) and downstream pathways are necessary.

### NADP(H) and redox rhythms

NADP(H) is an essential cofactor involved in maintaining redox homeostasis. The role of NADPH in oxidative stress response is well known, as it is the primary electron source for two of the most widely used antioxidant pathways—the glutathione and thioredoxin systems [24]. Specifically, NADPH donates two electrons to the oxidized forms of glutathione (GSSG) or thioredoxin (Trx-S_2_), becoming NADP^+^ yet generating reduced glutathione (GSH) or thioredoxin (Trx-(SH)_2_). GSH and Trx-(SH)_2_ are then used by their respective reductase enzymes to clear harmful oxidants from the cell [24, 39]. Inhibiting NADPH regeneration, via modulation of G6PD, NADK, or NOCT, has all been shown to lead to increased oxidative damage and cell death [70, 88, 89]. Interestingly, NAD(H) is also involved in oxidative stress response, yet its role is largely limited to restoring NADPH levels. Specifically, the activities of several NAD^+^-dependent enzymes that regenerate NADPH are upregulated in response to oxidative damage [90]. It has been shown that SIRT1 and SIRT5 deacetylate G6PD, and SIRT3 deacetylates IDH2 in an NAD^+^-dependent manner in order to increase cellular NADPH levels [91–93] (Fig. 2). Thus, while both cofactors have crucial functions in maintaining redox balance, the impact of NAD(H) is indirect and dependent on the activity of NADPH. Additionally, it is important to note that while NADP(H) levels can change in response to redox challenge, they are primarily controlled by the core circadian clock in the absence of secondary zeitgebers. This section will discuss how NADP(H) may participate in the circadian response to redox imbalance and how the changes in redox state can modulate clock activity through NADP(H).

#### NADP(H) and oxidative stress

Circadian variations in immune and oxidative stress responses are well established [94, 95]. As a clear exhibition of this interplay, it has been known for over 65 years that the lethality of lipopolysaccharide (LPS) injection differs significantly based on the time of injection. Specifically, lethality is higher when LPS is administered during the rest phase and lower when administered during the active phase [96]. With this, the profile of cytokines released in response to LPS differs based on the time of injection in both mice and humans [97, 98]. It was later found that this temporal effect is dependent on the core clock, for loss of *Bmal1* or *Reverbα* in murine macrophages eliminates the rhythmic response to LPS [98]. This may also be mediated in part by NADP(H), for mice lacking the NADP(H) phosphatase NOCT are more resistant to LPS challenge than wild-type (WT) mice [99]. Further, loss of NADK, the enzyme that catalyzes the reverse reaction of NOCT, has been found to blunt the pro-inflammatory response to LPS in murine macrophages [100]. Both NOCT and NAD(P)(H) rhythms are impacted by oxidative stress, and modulation of NOCT is associated with an altered inflammatory response [46, 101, 102]. Further studies are needed to determine whether the rhythmic response to LPS, or other modes of oxidative stress induction, is mediated by NADP(H) rhythmicity.

One study found that heart-specific overexpression of *Nampt* ameliorates the negative metabolic effects of diabetic cardiomyopathy. Specifically, *Nampt*-OX significantly decreases the GSSG/GSH ratio and attenuates the oxidation of thioredoxin substrates under high-fat diet (HFD). These effects are significantly reversed upon knockdown of *Nadk*. The authors also showed that *Nadk* knockdown significantly decreases NADPH levels yet has no effect on NAD^+^ or NADH levels. This, along with the NADPH dependence of both the GSH and thioredoxin antioxidant systems, suggests that the protective effect is primarily mediated by NADPH [103]. This coincides with the findings of another study that found that *Nadk2*-KO mice develop oxidative stress-induced hepatic steatosis [104]. As expected, *Nadk2*-KO mice have decreased levels of hepatic mitochondrial NADP(H) along with a higher GSSG/GSH ratio and increased ROS. Treatment with nicotinamide riboside (NR) restores hepatic NADP(H) levels and protects *Nadk2*-KO mice from developing steatosis [104].

#### NADP(H) and neurodegeneration

There have been several other studies demonstrating that disruption of core clock elements leads to disrupted inflammatory rhythms, and thus, worsened immune response [105–108]. Globally, this phenomenon is seen as shift workers have higher levels of white blood cells and C-reactive proteins in their plasma and an overall increased risk for inflammatory diseases [109–111]. Further, the risk for developing neurodegenerative disorders highly correlates with circadian disruption, and it is believed that this correlation is mediated by increased neuroinflammation and oxidative stress caused by clock disruption [112, 113]. Decreased expression levels of core clock genes as well as lower levels of NADP(H) and GSH have been found in the SCN of murine Alzheimer’s disease models [114]. Interestingly, a genome-wide association study found that a single nucleotide polymorphism (SNP) within *Noct* is highly associated with the risk of Alzheimer’s disease [115]. Additionally, high *Noct* expression levels were found in the dopaminergic neurons of postmortem brains from patients with Parkinson’s disease, suggesting that loss of rhythmic interconversion between NAD(H) and NADP(H) may play an important role in disease prognosis [116]. With this, it has been proposed that restoration of proper circadian NADP^+^/NADPH rhythms would slow the progression of Parkinson’s disease [117]. While no study to date has directly measured how NADP(H) rhythms are impacted by neurodegenerative disorders, evidence suggests that they may be valuable markers of inflammatory disease as well as potential therapeutic targets.

#### NADP(H) and the core clock

It is clear that the core clock regulates immune response, but there is increasing evidence that oxidative stress and cellular redox balance can reciprocally impact the activity of the core clock. Notably, it has been found that NADH and NADPH enhance the binding of both CLOCK:BMAL1 and NPAS2:BMAL1 heterodimers to DNA *in vitro* [118, 119]. The effect of NADPH is more pronounced than that of NADH, and interestingly, the oxidized cofactors NAD^+^ and NADP^+^ inhibit DNA binding, with stronger inhibition resulting from NADP^+^ [118]. While this suggests that NADP(H) pool may affect clock activation, the redox state appears to be the dominant factor affecting the complex binding. In fact, another study found that increasing extracellular pH increases the transcriptional activation of clock genes [119]. Other reports have found that 6-aminonicotinamide (6AN) treatment, which increases the cellular NADP^+^/NADPH ratio, enhances CLOCK:BMAL1 binding to DNA in U2OS cells [37]. Treatment with 6AN, as well as other established inhibitors of the NADPH-generating pentose phosphate pathway (PPP), also remodels circadian gene expression in U2OS cells, with both period lengthening and phase delaying effects [37]. While this and other studies suggested that the antioxidant regulator NRF2 (nuclear factor erythroid 2 (NF-E2)-related factor 2) is the mediator between redox imbalance and the core clock, others have found that loss of NRF2 has no effect on clock gene expression [37, 120, 121]. Instead, given that modulation of NADK expression also affects NRF2 expression, NADP(H) may be the principal metabolite connecting redox and circadian rhythms [55]. While the effect of NADK on the core clock has not been studied, it is known that NOCT, the enzyme that catalyzes the reverse reaction, has no impact on clock gene expression [122, 123]. Therefore, the exact impact of the total pool size or redox state of NADP(H) on the core clock remains elusive.

NADP(H) rhythms are primarily controlled by the core clock, for the expression of NAMPT, NADK, and NOCT are all regulated by this mechanism [23, 41, 59]. Despite this, NADPH rhythms persist in red blood cells, which lack a nucleus and the core circadian TTFL [35]. Other components of redox metabolism also exhibit circadian rhythmicity in enucleated cells; specifically, the levels of ATP and the oxidation state of peroxiredoxin oscillate under free-running conditions [35, 124]. Even though peroxiredoxin oxidation is independently rhythmic, their rhythms are altered in nucleated cells with a mutant clock [35, 125]. Reciprocally, circadian rhythms persist in mutant peroxiredoxin cyanobacterial strains, though the phase and amplitude are changed [125]. The impact of core clock mutation on NADP(H) redox rhythms has not yet been measured. Much like other redox systems, it is likely that NADP(H) acts as “accessory” rhythms. These “accessory” rhythms may have evolved independently of the core clock, but they are now dependent on such and serve to both reinforce and integrate core clock rhythms to peripheral metabolism.

### NADP(H) and metabolic rhythms

One of the most illustrious ways by which the circadian clock regulates metabolism is through the temporal control of NADP(H). In fact, it has been shown that the yeast metabolic cycle is dependent on the rhythmicity of NADP(H), and the PPP disruption completely abolishes metabolic cycling [38]. This likely translates to mammals where nearly 500 metabolic reactions require NADP(H) or NAD(H) and therefore are, at least indirectly, rhythmic, for their activity is dependent on the temporal availability of the necessary pyridine dinucleotide cofactors [23]. It is important to note, however, that while NADP(H) is primarily regulated by the core clock, which is supported by the evidence of altered NAD(P)(H) levels in clock mutant systems, its rhythmicity is also impacted by redox state and food intake [33, 34, 46]. As circadian disruption can be a product of activity and food intake occurring outside of the endogenous active phase, NADP(H) rhythms may be a key factor in the development of and prevention against circadian disruption-induced metabolic disease. With the rise in shift work and artificial light-induced circadian disruption, understanding this relationship could greatly impact the health of society [126]. This section will discuss how NADP(H) rhythmicity is impacted by metabolism, how NADP(H) in turn orchestrates metabolism, and how dysregulation of this relationship can lead to metabolic disease.

#### NADP(H) and diet

There is significant evidence that nutrients are a stronger zeitgeber for peripheral tissues than light [18, 19]. That is, circadian gene expression in peripheral tissues can be shifted by food intake independent of the SCN. The same is true for NADP(H), for both its overall concentration and redox ratio are impacted by nutritional status and may, in part, help entrain the core clock to such. However, the fields of calorie restriction, time-restricted feeding (TRF), and the like largely focus on how the effects are mediated by NAD(H) while overlooking that any impact on NAD(H) levels or rhythmicity likely directly affects that of NADP(H).

Numerous studies have found that cellular NADP(H) levels are sensitive to different diets. Specifically, one study found that a high-sucrose as well as a low-carbohydrate diet decreases the NADP^+^/NADPH redox ratio in rat liver tissue [127]. While the rhythmicity of NADP(H) in response to specific nutrients has not been measured, there are several reports demonstrating that NAD(H) rhythms are influenced by diet. A high-protein diet appears to sharpen the peaks of both cytosolic and mitochondrial NAD(H) rhythms in rat liver [34]. Additionally, it is well established that HFD feeding damps hepatic rhythms of both NAMPT and NAD^+^, as well as overall core clock gene expression [128–130]. These studies argue that the effect of HFD on circadian gene expression is mediated by loss of NAD^+^ rhythms, for supplementation with NAD^+^ or its precursor, nicotinamide mononucleotide (NMN), restores proper rhythmicity [128, 130]. However, another study found that hepatic NADP(H), not NAD(H), is significantly decreased under HFD conditions or type 2 diabetes induction. Further, this study argued that supplementation with NR, which ameliorates some of the negative metabolic effects of HFD, is likely working via increasing the NADP(H) pool rather than NAD(H) [131]. In support of this, reports have found that HFD perturbs the expression of both NOCT and NADK2 [123, 129, 132]. This highlights that the balance between NAD(H) and NADP(H), rather than just NAD(H) alone, could be responsible for the observed metabolic and circadian phenotypes under HFD conditions. More direct studies measuring NADP(H) rhythmicity in response to HFD are necessary to support this hypothesis.

Circadian gene expression in peripheral tissues is highly responsive to food intake, and misalignment between the core and peripheral clocks can lead to metabolic disease. Realignment of food intake to endogenous rhythms, as with TRF or fasting regimes, greatly increases metabolic health and longevity [133, 134]. Interestingly, TRF has been shown to restore metabolic rhythms even in clock-mutant mice [135]. This underscores the notion that, while the core clock regulates peripheral metabolic rhythms, metabolic rhythms can also be induced by rhythmic food intake. Even so, it is possible that NADP(H) mediates the entrainment of the peripheral clock to food intake. In fact, the same study that found TRF induces peripheral rhythms in clock mutants also found that TRF increases the amplitude of *Nampt* expression [135]. Other reports have found that not only the rhythmicity of *Nampt*, but also the rhythmicities of *Noct*, *Nadk2*, and NAD^+^ undergo phase shifts in response to TRF or fasting [33, 132, 136]. While the rhythmicity of NADP(H) under TRF has yet to be measured, it is known that periods of fasting can decrease the NADP^+^/NADPH redox ratios in mouse liver tissue [127, 137–140]. Additionally, TRF enhances the rhythmicity of enzymes involved in the PPP, which is the major cellular pathway that synthesizes NADPH [134, 139]. Given that the primary product of the PPP is NADPH, this could be another mechanism by which NADPH rhythmicity is reinforced. One study has even suggested that the PPP regulates core clock activity via regulation of the NADP^+^/NADPH redox ratio [37]. This would support previous reports that found that NADP^+^ and NADPH differentially regulate clock complex binding to DNA [118]. While this may be one means by which NADP(H) augments the core clock, others have found that increased activity of the sterol regulatory element-binding protein (SREBP) and ME1 disrupts NADP^+^/NADPH rhythms, which in turn leads to irregular sleep patterns in *Drosophila* [141].

While the role of NADP(H) is greatly understudied in this context, it is important to highlight the established roles that NAD(H) plays that are likely independent of NADP(H). For instance, it has been shown that liver-specific deletion of the NAD^+^-dependent PARP-1 can slow the entrainment of the peripheral clock to shifts in food intake [142]. Another study found that a TRF and calorie restriction regime increases hepatic NADH levels only during the subjective night. This rhythmic increase in NADH decreases BMAL1 chromatin binding via the inhibition of SIRT1 deacetylase activity by NADH and, in turn, produces rhythmic changes in downstream fatty acid and amino acid metabolism [139]. The notion that NAD(H) may impact the response of the clock to feeding is supported by other studies, for loss of *Sirt1* in VMH neurons also alters the response of circadian gene expression and behavior to TRF [143]. With this, *Sirt1*-KO mice are more prone to HFD-induced obesity and metabolic disease, while *Sirt1*-OX mice are protected from HFD-induced insulin resistance [144, 145]. This suggests that loss of communication between NAD(H) and the core clock could prompt circadian disruption-induced metabolic dysfunction.

Given that the rhythmicity of not only NAD(H), but also NAMPT, NOCT, and NADK, are affected by diet, the role of NADP(H) in entraining peripheral metabolism should be further investigated. Additionally, as both NAD(H) and NADP(H) rhythms are impacted by specific diet compositions, there could be a differential role of specific macronutrients at different circadian times of day. In summary, it is clear that any metabolic perturbations that alter NADP(H) rhythmicity impact downstream metabolic activity, but more evidence is needed to determine how such impacts overall circadian rhythms.

#### NAMPT and metabolism

As previously discussed, *Nampt* expression is clock-controlled, and its rhythmicity is lost in clock-mutant systems [41–43]. While loss of *Nampt* has no effect on circadian gene expression, it has significant effects on downstream metabolic rhythms. Specifically, one study found that liver-specific loss of *Nampt* significantly decreases fatty acid oxidation and oxygen consumption rate (OCR) [33]. A separate study found that adipose-specific loss of *Nampt* disrupts the rhythmicity of TCA cycle metabolites [45]. Given that loss of *Nampt* does not impact clock gene expression, these effects are likely due to loss of NAD(H) and NADP(H) rhythmicity. In fact, reports found that supplementation with NAD^+^ precursors, NR or NMN, especially administered at times in phase with endogenous NAD^+^ rhythms, rescues metabolic rhythms [33, 130, 146].

Interestingly, NAMPT has been implicated in metabolic disease in human populations. One report found that *Nampt* expression in adipose tissue is negatively correlated with body mass index (BMI) [147]. Another report found high expression of *Nampt* in the adipose tissue of obese populations, which was significantly lowered upon weight loss [148]. While studying global loss of *Nampt* is not possible due to *Nampt*^*−/−*^ embryonic lethality, *Nampt*^*+/−*^ mice exhibit no differences in body weight on either chow diet or HFD [103]. However, even without a difference in body weight, *Nampt*^*+/−*^ mice display impaired glucose tolerance and glucose-stimulated insulin secretion [149]. Further, *Nampt*^*+/−*^ mice have impaired diastolic function, reduced levels of the electron transport chain (ETC) complex proteins, and reduced NAD(H) and NADP(H) levels in heart tissue [103].

**Figure 3:**
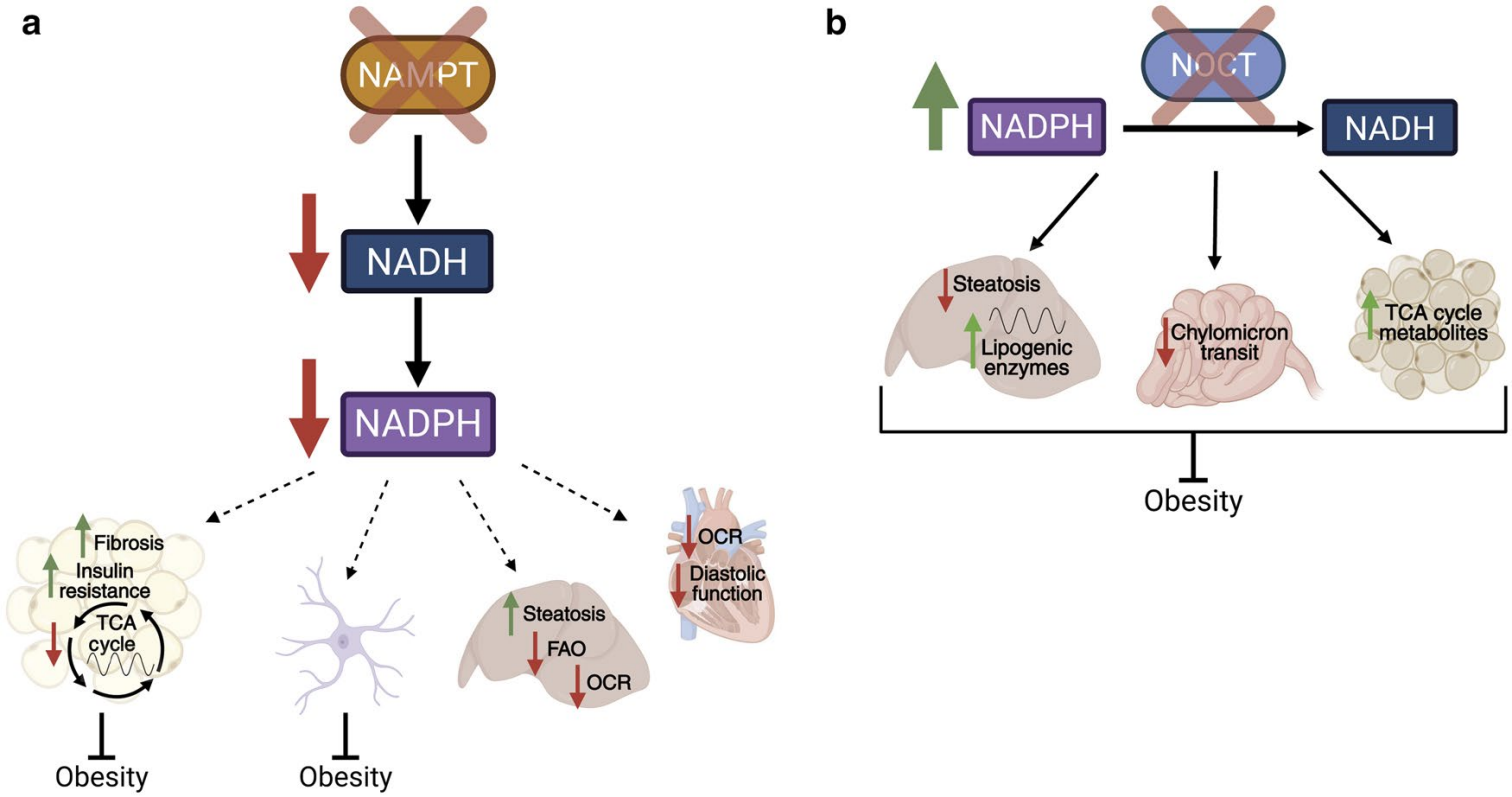
Metabolic phenotypes caused by disruption in NADP(H) rhythmic regulation. (a) Conditional loss (cKO) of *Nampt* within the adipose tissue leads to a diet-induced obesity (DIO)-resistant phenotype yet increased tissue fibrosis, insulin resistance, and decreased rhythmicity of TCA cycle metabolites. *Nampt*-cKO specific to astrocytes also leads to a DIO-resistant phenotype. *Nampt*-cKO specific to the liver and heart does not protect against DIO and displayes decreased OCR. *Nampt*-cKO in the liver also decreases fatty acid oxidation, which leads to increased steatosis. *Nampt*-cKO in the heart also decreases diastolic function. Dotted arrows depict tissue-specific KO. (b) *Noct*^*−/−*^ mice are resistant to HFD-induced obesity. Within the liver, *Noct*^*−/−*^ mice display resistance to HFD-induced hepatic steatosis and an increased amplitude of expression of lipogenic enzymes. Within the intestine, *Noct*^*−/−*^ mice display slow chylomicron transit, and within the BAT, they have higher levels of TCA cycle metabolites [150].

At a tissue-specific level, conditional loss of *Nampt* leads to unique and seemingly contradictory phenotypes [150] (Fig. 3). Mice with adipose-specific *Nampt* deletion are protected from both age- and diet-induced obesity, similar to what is seen in the human population [151, 152]. However, loss of *Nampt* in adipose tissue leads to adipose tissue fibrosis, insulin resistance, and low adiponectin [151–153]. Interestingly, astrocyte-specific deletion of *Nampt* also protects against HFD-induced obesity yet has no significant effect on glucose tolerance, insulin resistance, or adipose tissue fibrosis [154]. Further, loss of *Nampt* in the liver has no effect on body weight under HFD conditions, but exacerbates fat accumulation and liver damage [155]. These studies highlight not only the broad metabolic phenotypes caused by loss of *Nampt* but also the widespread, tissue-specific nature of NADP(H) metabolism. Even so, several studies have found an extracellular form of NAMPT (eNAMPT) in both murine and human plasma samples that significantly impacts NAD^+^ levels in several tissues throughout the body [149, 156, 157]. While the effect of eNAMPT on NADP(H) pools has not yet been measured, this adds another potential layer by which peripheral clocks communicate through NADP(H).

#### NADK and metabolism

Strong metabolic phenotypes are also observed when the rhythmic interconversion of NAD(H) and NADP(H) is disrupted. As previously discussed, NADK phosphorylates NAD(H) to generate NADP(H). There are several case reports of humans with mutations in the mitochondrial isoform, *NADK2*, that exhibit phenotypes similar to mitochondrial diseases. Specifically, patients have low mitochondrial NADP(H) levels along with high serum carnitine and lysine levels [158, 159]. In patient-derived fibroblasts, one study found that *NADK2* mutant cells have significantly lower OCR and higher extracellular acidification rate (ECAR), characteristic of ETC dysfunction [158]. Other reports have found that genetic variants that lead to decreased expression of *Nadk2* are associated with type 2 diabetes, non-alcoholic fatty liver disease, and hepatocellular carcinoma [160].

Studies have been able to phenocopy the disorders associated with *Nadk2* deficiency in humans with *Nadk2*-KO mice. One study found that *Nadk2*-KO mice have high serum carnitine and lysine and low mitochondrial NADP(H) [104]. This study also found that *Nadk2*-KO mice have significantly impaired hepatic fatty acid oxidation, resulting in hepatic steatosis and high circulating triglycerides under an atherogenic diet. Interestingly, even though these mice develop hepatic steatosis, both their body weight and composition do not differ significantly from WT controls [104]. A separate study also found that *Nadk2*-KO mice do not gain significantly more weight on an atherogenic diet than control mice; however, the *Nadk2*-KO mice gain more fat mass [160]. This study also observed impaired fatty acid oxidation, glucose intolerance, insulin resistance, and decreased mitochondrial NADP(H) levels in the *Nadk2*-KO mice [160]. These findings are in line with the previous study as well as human data. While the phenotypes from loss of *Nadk2* are consistent, there is still little consensus on the mechanisms eliciting such. Both reports found that *Nadk2* KO results in decreased activities of cAMP response element-binding protein H (CREBH), peroxisome proliferator-activated receptor alpha (PPARα), and PPARγ coactivator 1α (PGC1α)—transcription factors whose activities are impacted by the NAD^+^-dependent sirtuin enzymes [161, 162]. Thus, it is possible that loss of *Nadk2*, as well as subsequent changes in the mitochondrial NAD(H) pool, impairs the activities of CREBH, PPARα, and PGC1α and downstream lipid metabolism. Changes in NADP(H) also likely contribute to the phenotypes seen in *Nadk2*-deficient models. Human data found decreased activity of dieonyl-CoA reductase (DECR), an NADP(H)-dependent enzyme involved in the oxidation of polyunsaturated fatty acids, in patients with *Nadk2* mutations [158]. With this, the phenotypes observed in *Nadk2*-KO mice closely parallels those seen in *Decr*-KO mice [163, 164]. Further studies differentiating the effect of mitochondrial NAD(H) and NADP(H) levels on metabolic disease are necessary.

Loss of cytosolic NADK also elicits metabolic phenotypes. One study found that loss of NADK in *Drosophila* leads to reduced lipid stores in the *Drosophila* fat body as well as decreased mitochondrial mass [165]. These phenotypes are likely mediated by impaired fatty acid synthesis due to low cellular NADPH, for restoration of fatty acid synthesis increases both lipid stores and mitochondrial mass. Little is known about how loss of NADK in mammals impacts physiology, but given the phenotypes reported on NADK2, there are likely significant metabolic effects.

NADK activity has also been heavily implicated in cancer metabolism. As NADK is the only known enzyme that can generate *de novo* NADP^+^, studies have found that cancer cells upregulate *Nadk* expression to have more NADPH for antioxidant and anabolic pathways [166–168]. At the protein level, NADK activity is also regulated by growth signaling pathways, as protein kinase B (Akt) phosphorylates the amino-terminal domain of NADK, removing its self-inhibition [48]. This has made NADK a potential target for cancer therapeutics, for several studies have shown that NADK inhibition slows tumor proliferation [166, 167, 169]. Interestingly, while the majority of NADPH-dependent anabolic pathways occur in the cytosol and the cytosolic and mitochondrial NADPH pools are independently regulated, recent work has shown that NADK2 also contributes to cell growth [170, 171]. Specifically, one study found that NADK2 feeds proline synthesis for cell growth by generating pyrroline-5-carboxylate synthase, the enzyme in the bottleneck reaction of the proline synthesis pathway [170]. Thus, both the mitochondrial and cytosolic NADP(H) pools contribute to cell proliferation. It is well established that circadian disruption is a major risk factor for several types of cancer [172, 173]. It is possible that alterations in NAD^+^ rhythms and NADK activity is one of the mechanisms by which circadian disruption impacts cancer risk and progression.

#### NOCT and metabolism

Loss of the rhythmic NADP(H) phosphatase, NOCT, which catalyzes the reverse reaction to NADK, also prompts striking metabolic phenotypes (Fig. 3). Most notably, *Noct*-KO mice are resistant to HFD-induced obesity and hepatic steatosis, despite having similar food intake and activity to the WT controls [122]. This association is even seen in human populations, for genome-wide association study (GWAS) reports have found that NOCT expression significantly correlates with BMI [174, 175]. Additionally, *Noct* expression increases with years of shift work [175]. However, no changes in clock gene expression or circadian activity have been observed in *Noct*-KO mice, suggesting that circadian disruption impacts *Noct* expression, but NOCT does not directly impact the core clock [123].

There is evidence, however, that loss of NOCT impacts the rhythmicity of downstream metabolic pathways. For instance, *Noct*-KO mice display an increased amplitude of expression of lipogenic enzymes in their livers in the middle of their active phase (ZT18). Concordantly, *Noct*-KO mice have increased circulating triglycerides and very-low-density lipoproteins (VLDL) at ZT18 [123]. This apparent increase in lipogenesis is likely due to the increased cellular NADPH in the *Noct*-KO mice. While this is in line with the study that found that loss of NADK impairs lipogenesis, both *Drosophila* lacking NADK and mice lacking NOCT have decreased fat stores [165]. While logic would assume that loss of NOCT and NADK should elicit opposite phenotypes, it is possible that the phenotype is driven by disruption in NAD(P)(H) rhythmicity, rather than modulation of total levels. It is also likely, due to the ubiquitous nature of NAD(H) and NADP(H), that loss of NOCT causes numerous metabolic changes that may have contradictory effects. One study found that *Noct*-KO mice have increased amplitude of expression of *G6pdx* and *Me1* in their liver, and a separate study found that *Noct*-KO mice have increased TCA cycle metabolites in their brown adipose tissue (BAT) in response to cold stress [70, 176]. Thus, loss of NOCT has widespread tissue-specific effects.

In an effort to explain the lean phenotype caused by loss of NOCT, one study measured lipid absorption in *Noct*-KO and WT mice. While the *Noct*-KO mice appear to have impaired lipid absorption, with lower levels of radiolabeled lipids in their plasma post-gavage, there is no difference in fecal energy content between *Noct*-KO and control mice. In fact, the study found that *Noct*-KO mice have significantly higher lipid stores in their intestine post-gavage, suggesting an impairment in chylomicron transit rather than absorption [177]. As there are no obvious NADPH-dependent pathways in chylomicron packaging and secretion, the mechanisms behind this phenotype, as well as its relation to the lean phenotype on HFD, remain unknown. What is clear, however, is that loss of NOCT causes significant changes in lipid metabolism. Interestingly, this study also found that the lean phenotype on HFD is further exaggerated on a ketogenic diet yet less apparent on a high-carbohydrate diet [177]. This suggests that the fat content, not overall caloric content, is driving the phenotype. Studies examining how conditional loss of NOCT impacts tissue-specific metabolism are necessary to better understand the mechanism underlying the lean phenotype as well as the role of NADP(H) in tissue-specific metabolism.

## Conclusions and future directions

There is much still unknown about NADP(H) in the context of circadian metabolism. As discussed in this review, it is not currently known how core clock disruption impacts NAD(H) and NADP(H) rhythms. With this, it is not known how modulation of NAMPT, NOCT, or NADK affects NADP(H) rhythmicity. Given that disruption of any of these elements increases the risk for metabolic and neurodegenerative diseases, understanding whether and how NADP(H) rhythms are altered can help the field better understand the mechanisms behind and treatments for these diseases. Additionally, how NADP(H) rhythms are affected by different diets and dietary regimes, such as HFD and TRF, should be investigated. Understanding how NADP(H) responds to these central and peripheral zeitgebers will help further define the hierarchical structure of circadian rhythms.

Given the paucity of literature on the role of NADP(H) in lifespan, this review did not address the fields of aging and longevity. Because it has become evident that both circadian amplitude and NAD^+^ levels decrease with age, the field has remained largely focused on the role that the unphosphorylated nicotinamide plays in longevity [133, 178]. This focus is not unfounded, for there are several reports that provide evidence on how NAD^+^ impacts lifespan via mechanisms that are likely independent of NADP(H). For instance, it has been shown that the NAD^+^-dependent enzyme SIRT1 deacetylates both PER2 and BMAL1, and loss of SIRT1 in both the SCN and peripheral cells decreases the expression of core clock genes and the amplitude of activity [42, 146, 179]. It has also been found that SIRT1 activity and levels decrease in both the SCN and liver with age, and many of the negative age-related effects can be rescued with either *Sirt1*-OX or NR supplementation [146, 179]. It is, however, important to note the previously discussed report that found that NR supplementation has a greater effect on NADP(H) levels, underscoring that NADP(H) may play a role in some of the rescued phenotypes [131]. Additionally, it is important to highlight that NADP(H) likely impacts lifespan in manners independent of NAD(H). Oxidative stress is a major contributor to age-related diseases, so maintenance of high cellular NADP(H) and high amplitude NADP(H) rhythms, would likely help combat these negative effects. Future research studying the role of NAD(H) and aging should therefore also measure NADP(H) to delineate their effects. Further, research on the effects of NR, NMN, or other NAD^+^-precursor supplements on lifespan should do so in *Nadk*^*−/−*^ and *Nadk2*^*−/−*^ models to better understand whether the effects are due to increased levels of NAD(H) or NADP(H).

Lastly, in all of the research on NADP(H), it is clear that the cofactor has incredibly diverse, tissue-specific roles. Each tissue has a vastly different metabolism; thus, the primary pathways producing NADP(H) as well as the major pathways consuming NADP(H) differ across tissues. This is clearly evidenced by different metabolic phenotypes seen across different *Nampt* conditional knockout mouse lines. Further, seemingly contradictory phenotypes are seen across tissues within global *Noct^*−/−*^* mice. Though this work is laborious, it emphasizes that NADP(H) may be best studied in a tissue-specific manner to best understand its role in tissue-specific metabolism. With this, given the differences in rhythmicity between the mitochondrial and cytosolic isoform of NOCT, as well as between NADK and NADK2, it is likely that the rhythmic regulation of NADP(H) varies across cellular compartments. Further studies measuring whether NADP(H) rhythmicity differs among cellular fractions and which circadian element is regulating such rhythmicity is necessary.

In conclusion, it is clear that NADP(H) plays a vital role in regulating circadian metabolism. As NADP(H) has been largely underappreciated within the circadian, metabolism, aging, and adjacent fields, this review aims to highlight the striking effects caused by altering NADP(H) levels, redox state, and rhythmicity. The complex spatiotemporal regulation of NADP(H) likely develops as an evolutionary safeguard to the increasing number of external stimuli that differentially affect peripheral and central metabolism. Future studies on the role of NADP(H) in circadian metabolism will offer valuable and exciting information that will both expand and connect fields of research.
